# The Evaluation of Physical Performance in Rowing Ergometer: A Systematic Review

**DOI:** 10.3390/jfmk10040437

**Published:** 2025-11-09

**Authors:** Iñigo Borges, Santiago Veiga, Pablo González-Frutos

**Affiliations:** 1 Faculty of Health Sciences, Universidad Francisco de Vitoria, 28223 Madrid, Spain; inigo.borges@ufv.es; 2 Sports Department, Universidad Politécnica de Madrid, 28040 Madrid, Spain; santiago.veiga@upm.es

**Keywords:** rowing performance, rowing ergometer, 2000 m test, critical power, 3 min all-out test

## Abstract

**Background**: Growing interest in optimizing rowing performance has led to numerous ergometer-based testing protocols. However, this diversity has created a lack of consensus on which physiological variables best predict rowing performance. This systematic review provides an updated synthesis of the main ergometer testing protocols and identifies the variables most strongly associated with 2000 m performance. **Methods**: A systematic search was conducted across PubMed, Web of Science, and Scopus databases, following PRISMA and STROBE guidelines. Studies were selected based on predefined inclusion criteria, and methodological quality was assessed accordingly (PROSPERO: CRD420251027702). **Results**: Thirty-four studies comprising 909 rowers (657 men, 252 women) across elite (20%), sub-elite (32%), and recreational (47%) levels were analyzed. The 2000 m test was the most frequently employed protocol (79%), followed by incremental (INCR) tests. The 2000 m test reflects competition performance, whereas INCR tests are primarily used to assess VO_2_max and PPO, the variables most strongly correlated with 2000 m outcomes. Power at lactate threshold and CP also showed strong associations with performance, particularly when measured through short, time-efficient protocols that minimize fatigue. **Conclusions**: The strongest and most consistent correlates of 2000 m ergometer performance are VO_2_max and PPO (r = 0.83–0.99). CP is likewise strongly associated (*n* = 4 studies) but rests on a smaller evidence base. Given that 72% of the analyzed sample comprised male participants, extrapolation of these findings to female rowers warrants caution.

## 1. Introduction

The rowing ergometer is a tool that allows the simulation of the rowing movement and the practice of rowing outside the aquatic environment. Given the ease of controlling the training volume and evaluating physical performance [1], it provides a standardized and reproducible testing environment, independent of external factors such as weather or water conditions [2]. It allows precise monitoring of physiological responses and mechanical output, which makes them highly practical for both scientific research and daily training applications.

Different brands of rowing ergometers have been developed, such as Gjessing or RowPerfect, but the most marketed model is the Concept2, which has held a leading position in the market since the introduction of the Model IIb in 1986 [3]. Subsequently, Concept2 designed new models with some technical improvements in terms of user comfort, durability, and monitor display, although neither the biomechanics of movement nor the physical evaluation were modified from the previous models [4]. Currently, athletes and practitioners can visualize in real time different variables related to practice, schedule workouts, and synchronize with various devices [3] while controlling the drag factor that increases or reduces resistance depending on the air output. The main variables are the average power (AP) in (W) and the split time (per 500 m), and the stroke frequency (strokes per minute). Data from the IIb model have been compared with mechanical sensors, showing a nearly perfect correlation (r = 0.96) and therefore a high reliability [5].

One of the main sports applications of the rowing ergometer is the assessment of physical performance in teams or groups of athletes [3], as the conditions of the tests can be easily standardized regardless of environmental conditions or water [6]. The most common tests used are (i) distance test, (ii) time test, (iii) maximum stroke test, and (iv) both continuous and discontinuous INCR tests. For distance tests, the 2000 m test is the most common as it represents the distance used in competition according to the rules of the Fédération Internationale des Sociétés d’Aviron (FISA). However, shorter distances such as 500 m and 1000 m [7] are also used to calculate CP using the traditional model. Additionally, da Silva et al. [8] employed the 100 m test to predict 2000 m performance, reporting a correlation of r = 0.73. Longer distances, such as 6000 m are also employed to assess aerobic fitness [9], showing strong correlations with performance variables, including power output (PO) at the ventilatory threshold (r = 0.74) and at VO_2_max (r = 0.75).

Although researchers have also aimed to predict 2000 m performance using shorter-duration tests such as the Wingate test [10,11,12], the 20 s test [13,14], or the 60 s test [14,15], reported correlations vary substantially depending on protocol and athlete level. In particular, correlations with 2000 m performed range from r = 0.79–0.87 for the Wingate test (large) to r = 0.92–0.94 for the 60 s test (nearly perfect), with differences primarily attributable to competitive level and the degree of protocol standardization. The Wingate test, traditionally performed over 30 s, has shown moderate to strong correlations with 2000 m rowing performance, particularly when mean PPO is used as a predictor10. Similarly, the 20 s all-out test has demonstrated significant associations with both AP and PPO during longer efforts, making it a practical and time-efficient alternative for estimating rowing capacity [14,15]. More recently, Cerasola et al. [14,15] reported that the 60 s test provides a stronger predictive value compared to shorter tests, with correlation coefficients reaching up to r = 0.90 when analyzing mean PPO and pacing consistency throughout the trial.

Maximum stroke tests focus on evaluating peak power in a small number of rowing strokes. For instance, 1-stroke tests [16] show a moderate correlation with 2000 m performance (r = 0.62), while the 5-stroke [1,17] and 10-stroke protocols [18] are also used. Peak power is 9% higher in elite than in amateur rowers, highlighting the relevance of this variable in high-level performance.

The incremental (INCR) test is commonly used to evaluate the physiological response to progressive workloads on the rowing ergometer [1,19,20]. Among studies employing INCR test protocols (*n* = 29), the continuous variant is frequently used to determine maximal oxygen uptake (VO_2_max), which shows strong correlations with the average power of the 2000 m test (r = 0.76–0.99) [1,19,20]. However, VO_2_max does not consistently exhibit higher correlations than peak power output (r = 0.83–0.99) [19,21], indicating that both variables similarly reflect aerobic performance in rowing. The incremental test can be performed in continuous or discontinuous format, depending on the objectives of the evaluation and the protocol design. In either case, INCR protocols effectively capture aerobic capacity, and both maximal and submaximal indices (VO_2_max and PPO) should be interpreted together when evaluating performance potential.

In the discontinuous INCR tests, rowers commonly perform 3 min rowing bouts with a 1 min rest period, with an intensity increase that varies depending on the level of participants and gender [22]. An alternative approach is the continuous INCR test, where intensity is increased every minute with no effort interruptions and, therefore, in a shorter amount of time. This is the most widely used test to measure maximal oxygen uptake (VO_2_max), which shows the largest correlation with the average power (AP) of the 2000 m test [23].

Critical power (CP) is an increasingly important variable for assessing rowing performance, representing the asymptote of the power–duration relationship that separates the heavy and severe exercise domains [24]. Across studies, CP has been estimated using three main approaches: (i) two-parameter linear models (work–time or power–1/time formulations) [7,25], (ii) three-parameter hyperbolic models [7,25], or (iii) the 3 min all-out test [16,26], in which end power (average power over the last 30 s) approximates CP (r = 0.75). Slight discrepancies between model equations may lead to minor variations in CP values, limiting direct comparison between studies.

Given the variety of tests and protocols used in rowing performance evaluation, it is essential to clarify which test or variable among those proposed and their relationships with 2000 m performance, thereby establishing a foundation for future meta-analytic work in this domain. Therefore, the aim of the present study was: (1) to provide an updated review of the existing tests for physical evaluation in rowing ergometers and (2) to synthesize the main variables measured in each test and their relationships with 2000 m performance.

## 2. Materials and Methods

The present systematic review was conducted according to the Preferred Reporting Items for Systematic reviews and Meta-Analyses (PRISMA) [27] checklist, and the PROSPERO guidelines (registration no. CRD420251027702).

### 2.1. Systematic Literature Search

A literature search was conducted using the PubMed, Web of Science, and Scopus databases. The strategy for searching in the databases was carried out using terms grouped into two search strings. The search syntax was adapted for each database:

(1) “rowing ergometer” or “rowing test” or “rowing ergometry” or “indoor rowing” and “power output” or “critical power” or “all-out”; (2) “rowing ergometer” or “rowing test” or “rowing ergometry” or “indoor rowing” and “reliability” or “validity” or “performance” or “test”.

Filters: humans, original peer-review articles, no restriction on sex, competitive level, or language search stage. Reference lists were hand-searched.

All articles were selected based on eligibility criteria. Additional records were identified through other sources (hand searching the reference list of articles). Within the article, the characteristics of the level of the participants, tests used, experimental designs, description of the intervention, and the results of the main variables (CP, aerobic and anaerobic threshold, lactate threshold, and ventilatory threshold) with the correlation of the different tests used were classified.

### 2.2. Inclusion and Exclusion Criteria

Original scientific research based on predictive tests of performance or variables relevant to performance in humans was included. Studies published up to December 2022 were included in the analysis.

According to the PICO(s) strategy for searching, the inclusion criteria were as follows: (1) Population. All articles that performed the tests on rowers (women and men), adolescent and adult rowers (>13 years) with competitive experience at any level (Class A–C). (2) Intervention. The tests were carried out only on the Concept2 rowing ergometer. (3) Comparison. Recording of variables that are especially important in the performance of rowers such as maximum oxygen consumption, AP, c, and aerobic and anaerobic threshold. (4) Outcomes. Average or maximum W on AP in the INCR test correlates with the main physiological variables related to performance in the rowing ergometer.

Exclusion criteria were (1) performed tests on other rowing ergometers such as the RowPerfect or Gjessing, (2) used supplementation; (3) inclusion of rowers with physical and/or intellectual disabilities; (4) the relationship between performance and hormonal responses is sought; (5) calculate biomechanical variables but not performance predictors; (6) compare results with on-water performance.

### 2.3. Data Extraction Strategy

**Figure 1:**
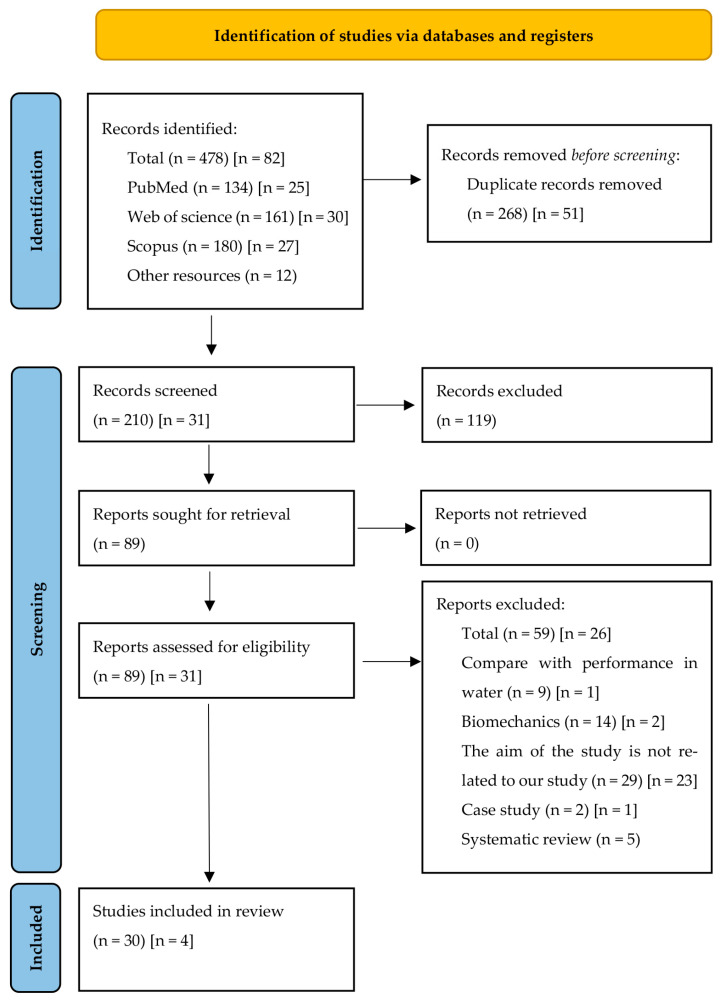
Flow diagram for screening and selection studies according to Preferred Reporting Item for Systematic Reviews and Analysis (PRISMA).

The initial literature search was conducted in December 2022, followed by a second search in June 2023. A final update was performed in October 2025 to ensure comprehensiveness and capture any relevant studies published during the time elapsed since the initial search. In Figure 1, the June 2023 search is shown in parentheses, whereas the October 2025 update is indicated in brackets. The authors conducted a peer review of the articles to ensure the reliability and eligibility of the selection of articles for the review, according to the criteria for preparing systematic reviews (PRISMA) [27]. On 24 October 2025, an update to the search process was carried out using the same methods as in the first search. Data extraction was conducted independently by two reviewers (IB and PG-F) using a piloted, standardized spreadsheet (Microsoft Excel 2019), with a third author (SV) arbitrating disagreements. We extracted sample characteristics: (A) author(s); (B) characteristics of the sample (n, sex, and age); (C) competitive level; (D) tests and protocols used in the experimental design (INCR test, fixed distance test, and time-determined test) such as the tools used (gas analyzers, lactic acid analyzer and ergometer data recording programmes); (E) main results of the studies (reliability of evidence, reproducibility and validity).

### 2.4. Study Quality Assessment

The Strengthening the Reporting of Observational Studies in Epidemiology (STROBE) checklist was used to determine the quality of the studies [28]. The checklist was composed of 22 items clustered into six categories belonging to the different study sections: Title and Abstract (item 1), Introduction (items 2 and 3), Methods (items 4–12), Results (items 13–17), Discussion (items 18–21), and Funding (item 22). A score of ‘0’ was assigned to incomplete items or items with a lack of information, and ‘1’ to items that were described accurately. The overall rating obtained from the sum of the item values was categorized according to following levels: very low quality (0–4 points); low quality (5–8 points); medium quality (9–12 points); high quality (13–16 points); and very high quality (17–22 points). The study quality assessment was carried out by two independent reviewers (IB and PG-F). Inter-rater agreement was high (initial agreement: 91%; κ = 0.84), with disagreements resolved through discussion with a third reviewer (SV).

### 2.5. Risk of Bias

The risk of bias assessment followed the approach described by Kilbey et al. [29], which was considered the most appropriate given the scope and characteristics of the included studies. Five criteria were evaluated (Table 1): (A) peer-reviewed publication, (B) sample size, (C) population definition (age, sex, sport, competitive level, and experience), (D) methodological clarity, and (E) statistical test reporting. Each criterion was rated on a 0–2 scale, except for criterion (E), which was binary. Total scores were used to classify studies as Good (≥7 points), Fair (4–6 points), or Poor (≤3 points). The assessment was independently conducted by two reviewers (I.B. and P.G.-F.), and any disagreements were resolved through discussion with a third reviewer (S.V.).

### 2.6. Data Synthesis and Analysis

Pearson correlation coefficients between physiological variables and 2000 m ergometer performance were extracted from each study for comparison across athlete levels and testing protocols. The magnitude of correlation coefficients was interpreted according to the scale proposed by Hopkins [31]: r < 0.10, trivial; 0.10–0.29, small; 0.30–0.49, moderate; 0.50–0.69, large; 0.70–0.89, very large; >0.90, nearly perfect; and 1.00, perfect.

## 3. Results

### 3.1. Review Statistics

The four stages of the search process are shown in Figure 1. (1) Identification: The first author (IB) identified scientific studies through a single search process (*n* = 560), including records from databases and registers. (2) Screening: The second author (SV) removed duplicate records (*n* = 319), and the first author (IB) excluded those not relevant through a preliminary reading of the title, abstract, and keywords (*n* = 119). (3) Eligibility: The authors IB and PG-F assessed the remaining full-text reports and excluded those unrelated to the topic according to the exclusion criteria (*n* = 85). (4) Inclusion: Finally, the remaining studies were included in the systematic review (*n* = 34).

### 3.2. Study Characteristics

The distribution of male and female rowers across competitive classes is summarized in Table 2. In total, 909 athletes (657 men and 252 women) were analyzed, representing 72% men (95% CI: 69–75%) and 28% women (95% CI: 25–31%). Most studies were conducted in male samples (*n* = 23), six in female samples, and five included both sexes. Following the classification proposed by Lawton et al. [32], five studies analyzed elite rowers (Class A), seven examined sub-elite athletes (Class B), and 18 focused on non-elite or university-level rowers (Class C). Four studies further subdivided participants into heavyweight (HWT) and lightweight (LWT) categories [1,19,22,23]. Finally, three articles included mixed-level simples [7,18,33]. Detailed participant characteristics and study aims are presented in Table 3, arranged chronologically to facilitate the retrieval of information by researchers and coaches regarding rower level and sex.

Reviewing the objectives of the articles (Table 3), we have made a classification of three different objectives. 22 articles aim to know the physiological variables (aerobic and anaerobic capacity, lactate thresholds and VO_2_max) that are related to performance in the 2000 m test [1,7,9,18,19,20,22,23,25,33,35,37,38,40,41,42,43,45,46,47,48], five articles aim to develop mathematical models to predict performance in ergometer rowing [8,10,12,21,44], and seven articles aim to know the relationship between the AP of the 2000 m test with the short-duration tests [11,13,14,15,16,26,39].

### 3.3. Main Tests for Performance Measurement

Table 4 and Table 5 summarize the distance or time tests, and the results are represented with the total time to complete the test or with the AP. The test that has been performed more times by the researchers was the 2000 m test (*n* = 27) [1,7,8,10,12,13,14,15,16,18,19,25,26,28,29,30,31,32,33,34,38,39,40,44,45,46,47,48], that also represents the competition distance. Table 6 and Table 7 include the articles that have performed INCR tests, both continuous and discontinuous. The results summarize the maximum power recorded in the test and the VO_2_max. This table also shows the main findings of the research and the correlation between principal variables measured. The results of the INCR tests are very important, as this is the second most widely used test in research, with the discontinuous test being used 16 times [1,7,9,12,18,20,21,22,26,33,34,41,44,45,47,48] and the continuous test 13 times [1,7,13,14,15,20,23,37,38,39,40,42,43]. The next most frequently used tests are the distance tests. In this case, we will break down the number of times they have been performed: 100 m (*n* = 1) [8], 500 m (*n* = 2) [7,25], 1000 m (*n* = 1) [7], 1500 m (*n* = 2) [11,45] and 6000 m (*n* = 4) [7,9,16,25]. In the case of time tests, the 20 s (*n* = 2) [13,14], Wingate (*n* = 3) [10,11,12], 60 s (*n* = 2) [14,15], 3 min all-out (*n* = 2) [16,26], 6 min all-out (*n* = 1) [43] and 20 min (*n* = 1) [18] tests were used. In four of the studies, maximal effort tests were conducted using maximal stroke test to determine peak PO, the assessment protocols included tests of 1, 5, 6 and 10 strokes [1,16,17,18,46]. The CP or critical CV was calculated on four articles [16,25,26,39].

Among the key variables reported, three primary metrics were analyzed across competitive levels: VO_2_max, PPO achieved in an INCR test, and AP during a 2000 m trial [1,19]. Secondary physiological indicators included blood lactate concentration at the anaerobic threshold [1,18], CP [25,26,39], and ventilatory threshold, which were used in several studies to further characterize endurance capacity and metabolic efficiency [1,49]. The results are organized by competitive level (Classes A, B, and C) and by sex, as summarized in Table 4. Overall, the data show a progressive decline in both VO_2_max and power outputs from elite to recreational rowers. The largest number of participants corresponded to Class C athletes, reflecting the predominance of studies conducted with non-elite or university-level rowers. Sub-elite samples (Class B) displayed greater variability, likely due to transitional training statuses and heterogeneous testing protocols. Differences between men and women were consistent with known physiological determinants, with female rowers exhibiting lower absolute but comparable relative performance values. These findings highlight the key role of aerobic and power capacities as distinguishing factors across competitive levels.

### 3.4. Main Findings

The shorter time tests were performed in 11 articles and showed large correlations with 2000 m performance (r = 0.73–0.94), providing useful information at specific points of the season to track rowers’ improvement. Within this group, we observed that the 20 s and 60 s tests have a nearly perfect relationship with the AP of the 2000 m test (r = 0.92–0.94) [13,14,15], unlike the Wingate (r = 0.79–0.87) [10,11,12] and the 100 m test (r = 0.74) [8], which showed a very large correlation.

Table 6 summarizes the results of the discontinuous INCR tests (*n* = 16) and Table 7 results of the continuous INCR tests (*n* = 13) reported in articles. In four articles, both tests (INCR test continuous and discontinuous) were performed [7,20,23,40], with the aim of determining which of the measured variables were strongly associated with performance in the 2000 m test. Among the most important findings (Table 6 and Table 7), we can highlight the relationship between the main physiological variables with 2000 m performance. The physiological variable with the largest correlation is VO_2_max (r = 0.61–0.99) [1,17,19,20,22,23,33,37,38,39,41,42,48], followed by power at lactate threshold (r = 0.73–0.92) [12,20]. Maximum power in the INCR tests was also highly relevant, given its very large to nearly perfect association with 2000 m performance (r = 0.83–0.98) [1,19,22,33,41,47].

### 3.5. Study Quality

The quality analysis (RAE–Performance Strengthening the Reporting of Observational Studies in Epidemiology (STROBE) checklist) [28] yielded the following results: (a) The quality scores ranged from 15 to 21. (b) The average score was 18 points. (c) A total of 10 out of the 30 included studies (30%) were categorized as high quality (13–16 points), and 24 (70%) were considered very high quality (17–20 points). The highest scores were obtained in the Title and Abstract (100%), Introduction (98.5%), Results (82.9%), Method (81.4%), and Discussion (79.4%) sections. The lowest scores were detected in the other section (32.4%). Among the highest-quality studies, items 1 (Title and abstract), 2 (background/rationale), 4 (Study design), 6 (participants), 12 (Statistical methods), 16 (main results), and 18 (key results) were considered complete (100%). By contrast, the most uncommon items were numbers 19 (Limitations) with 33.3%, 17 (other analyses) with 30.0%, 22 (Funding) with 33.3%, and 9 (Bias) with 0%.

### 3.6. Risk of Bias

**Figure 2:**
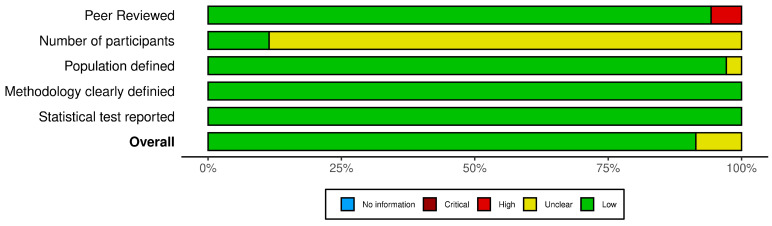
Risk of bias [30].

Using the Kilbey et al. [29] criteria, overall study quality was predominantly Good, with the remainder rated Fair; no study was classified as Poor. As shown in Figure 2, most studies demonstrated a low risk of bias, particularly regarding methodological clarity and statistical reporting, whereas uncertainty was mainly observed in the number of participants and population definition criteria. The most frequent downgrades occurred for Population defined (incomplete reporting of sex, competitive level, or experience) and Statistical test reported (absence of significance or uncertainty for correlations), mirroring reporting gaps noted in prior reviews. Disagreements between the two reviewers were resolved by consensus.

**Table 6:** Most important variables of the discontinuous INCR test and main results of the studies.

Author	Discontinuous INCR Test		Main Findings
	Maximal Power	VO_2_max	
Astridge et al. [45]	NR	NR	Maximal power of 1500 m was 5.2% higher than 2000 m (*p* < 0.01)
Bourdin et al. [19]	441.6 ± 33.9	5.68 ± 0.32 L·min^−1^	PPO in INCR test correlated with 2000 m AP (r = 0.92; *p* < 0.0001), VO_2_max correlated with 2000 m AP (r = 0.84; *p* < 0.0001) and with INCR test PPO (r = 0.84; *p* < 0.0001)
Bourdin et al. [22]	278 ± 29	3.68 ± 0.30 L·min^−1^	PPO in INCR test correlated with 2000 m AP (r = 0.88; *p* < 0.001), VO_2_max correlated with 2000 m performance (r = 0.83; *p* < 0.001) and with INCR test PPO (r = 0.81; *p* < 0.001)
Bourdon et al. [48]	MP: 286.7 ± 16.8	4.23 ± 0.22 L·min^−1^	2000 m AP correlated with INCR test disc. VO_2_max (r = 0.99; *p* = 0.22)
Cheng et al. [26] (CP test)	NR	NR	3 min all-out correlated with CP (r = 0.745; *p* < 0.05), 3 min all-out correlated with VO_2_max (r = 0.664, *p* < 0.05)
Cosgrove et al. [20]	NR	NR	VO_2_max correlated with 2000 m velocity (r = 0.85; *p* < 0.001); PPO at lactate threshold correlated with 2000 m velocity (r = 0.73; *p* < 0.004)
Ingham et al. [23]	PO at VO_2_max:	4.62 ± 0.82 L·min^−1^	2000 m power correlated with INCR test cont. Maximum minute power (r = 0.98; *p* < 0.05), VO_2_max correlated with the power associated with VO_2_max in INCR test disc. (r = 0.90; *p* < 0.05) and VO_2_max of INCR test cont. (r = 0.88; *p* < 0.05)
	285.0 ± 44.5		
Izquierdo-Gabarren et al. [18]	PO at 4 mmol·L^−1^:	NR	Power at 4 mmol·L^−1^ correlated with 20 min (r = 0.65; *p* < 0.01) and with 10 maximal strokes power (r = 0.5; *p* < 0.05)
	253.2 ± 34		
Jensen et al. [47]	350 ± 65	4.81 ± 0.78 L·min^−1^	2000 m AP correlated with INCR test disc. Maximum power (*p* < 0.001) and 2000 m VO_2_max correlated with INCR test disc. VO_2_max (*p* = 0.51)
Mazza et al. [21]	T: 843 ± 57 s	3.50 ± 0.60 L·min^−1^	Predicted and measured VO_2_max had a high correlation in female rowers (r = 0.97; *p* < 0.001)
McGrath et al. [44]	286 ± 77	NR	The estimated FTP with INCR test disc. test correlated with calculated in a 20 min test (r = 0.98)
Messonnier et al. [33]	NR	5.32 ± 0.14 L·min^−1^	INCR disc. test power correlated with 2000 m AP (r = 0.73; *p* < 0.001) and INCR disc. test VO_2_max correlated with 2000 m VO_2_max (r = 0.54, *p* < 0.01)
Mikulic [9]	PO at VO_2_max:	5.50 ± 0.30 L·min^−1^	INCR disc. power at VO_2_max and 6000 m power at VO_2_max (r = −0.732; *p* < 0.01) and INCR disc. power and 6000-m time (r = −0.484, *p* < 0.05)
	423.8 ± 38.1		
Possamai et al. [7]	308 ± 37	4.19 ± 0.39 L·min^−1^	CP correlated with MLSS (*p* < 0.001). 500 m AP with MLSS (r = 0.65), 1000 m AP with MLSS (r = −0.86), 2000 m AP with MLSS (r = 0.78) and 6000 m AP with MLSS (r = 0.39)
Riechman et al. [12]	PO at LT: 138 ± 27.2	3.18 ± 0.35 L·min^−1^	Wingate AP correlated with 2000 m AP (r = −0.870; *p* < 0.001) INCR disc. test power at LT correlated with 2000 m AP (r = −0.822; *p* < 0.001) and INCR test disc. VO_2_max correlated with 2000 m AP (r = 0.502)
Turnes et al. [41]	284.8 ± 44.7	4.61 ± 0.62 L·min^−1^	INCR disc. test PPO correlated with 2000 m AP (r = 0.978; *p* < 0.01) and INCR disc. test VO_2_max correlated with 2000 m AP (r = 0.883; *p* < 0.01)

Abbreviations: NR; not reported.

**Table 7:** Most important variables of the continuous INCR test and main results of the studies.

Author	Continuous INCR Test		Main Findings
	Maximal Power	VO_2_max	
Cataldo et al. [13]	NR	4.62 ± 0.66 L·min^−1^	2000 m AP correlated with 20 s AP (r = −0.947; *p* < 0.001) VO_2_max correlated with 2000 m AP (r = −0.884; *p* < 0.0001)
Cerasola et al. [14]	NR	4.66 ± 0.84 L·min^−1^	2000 m AP correlated with 60 s AP (r = −0.943; *p* < 0.0001) and with VO_2_max (r = −0.761; *p* < 0.0001)
Cerasola et al. [15]	NR	NR	2000 m AP correlated with 60 s AP (r = −0.914; *p* < 0.0001) and 20 s AP (r = −0.920; *p*< 0.0001)
Cosgrove et al. [20]	NR	4.5 ± 0.4 L·min^−1^	VO_2_max correlated with 2000 m velocity (r =0.85; *p* < 0.001); PPO at lactate threshold correlated with 2000 m velocity (r = 0.73; *p* < 0.004)
Gillies and Bell [37]	NR	3.52 ± 0.84 L·min^−1^	2000 m time correlated with VO_2_max (r = 0.96, *p* < 0.05) and with PPO at VO_2_max (r = 0.83; *p* < 0.05)
Huerta Ojeda et al. [42]	PO at VO_2_max:	4.07 ± 0.26 L·min^−1^	VO_2_max in INCR test and 2000 m have high correlation (*p* < 0.05)
	334.7 ± 20.2		
Huerta Ojeda et al. [43]	PO at VO_2_max:	4.09 ± 0.26 L·min^−1^	VO_2_max in INCR test correlated with 6 min VO_2_max (*p* = 0.16), maximal aerobic power correlated with de PPO at VO_2_max in 6 min test (*p* = 0.0001) and AP (*p* = 0.004)
	325.54 ± 39.82		
Ingham et al. [1]	NR	NR	2000 m performance correlated with power of 5 Maximal Strokes (r = 0.95; *p* < 0.001) with VO_2_max (r = 0.93; *p* < 0.001) and with the power in 4 mmol·L^−1^ of the INCR test (r = 0.92; *p* < 0.001)
Ingham et al. [23]	MMP: 352.0 ± 68.2	4.67 ± 0.85 L·min^−1^	2000 m power correlated with INCR test cont. Maximum minute power (r = 0.98; *p* < 0.05), VO_2_max correlated with the power associated with VO2max in INCR test disc. (r = 0.90; *p* < 0.05) and VO_2_max of INCR test cont. (r = 0.88; *p* < 0.05)
Kendall et al. [39]	261 ± 27	3.14 ± 0.31 L·min^−1^	2000 m performance correlated with CV (r = 0.886; *p* < 0.001) and VO_2_max correlated with 2000 m performance (r = −0.923, *p* < 0.001)
Otter et al. [40]	290 ± 44	5.3 ± 0.4 L·min^−1^	The SmRT was able to accurately predict 2000 m rowing time when performed on an indoor rowing ergometer. Stage 1 power (70% of HRmax) with 2000 m (r = −0.73), Stage 2 power (80% of HRmax) with 2000 m (r = −0.85) and Stage 3 power (90% of HRmax) with 2000 m (r = −0.93).
Possamai et al. [7]	311 ± 35	4.08 ± 0.47 L·min^−1^	CP correlated with MLSS (*p* < 0.001). 500 m AP with MLSS (r = 0.65), 1000 m AP with MLSS (r = −0.86), 2000 m AP with MLSS (r = 0.78) and 6000 m AP with MLSS (r = 0.39)
Shimoda et al. [38]	NR	4.1 ± 0.4 L·min^−1^	VO_2_max correlated with 2000 m time (r = − 0.61; *p* = 0.012)

Abbreviations: NR; not reported.

## 4. Discussion

The present systematic review aimed to synthesize the available protocols for physical evaluation on Concept2 rowing ergometers. VO_2_max and PPO derived from INCR test were the most frequently assessed variables, both showing strong associations with 2000 m performance. PPO at lactate threshold and CP also demonstrated very large correlations with performance, particularly when evaluated through short-duration, time-efficient protocols that minimize fatigue. These findings are consistent with previous evidence highlighting the predictive value of VO_2_max and power-related measures in rowing performance, while also suggesting that submaximal indices can provide practical alternatives in monitoring frameworks. Nevertheless, the strength of these associations appears to vary according to competitive level, which warrants further research to clarify how athlete status may mediate the predictive utility of different testing protocols.

### 4.1. Study Characteristics

The predominance of male participants (about 70%) and recreational-level samples (around 56%) limits the generalizability of current findings to elite female rowers. Only six studies focused exclusively on women [12,16,21,22,34,39], and just four are distinguished between heavyweight and lightweight categories [1,19,22,23], potentially masking relevant metabolic and physiological differences across weight classes defined by FISA regulations. This underrepresentation limits the development of sex-specific and weight-class-specific normative data, which are essential for individualized training prescription, performance benchmarking, and talent identification in these populations. Most investigations targeted physiological determinants of performance, with few addressing predictive modelling or short-duration test correlations, reflecting a narrow methodological scope. Collectively, these patterns highlight the need for future research to include more diverse and high-performance samples, as well as more integrative analytical approaches.

### 4.2. Main Tests for Performance Measurement

The 2000 m test was prioritized as it represents the standard competition distance and the most frequently studied protocol (Table 8), allowing direct comparability across cohorts and consistent benchmarking of VO_2_max and PPO relationships. Although alternative tests (e.g., 6000 m, 20 s, 60 s, or 3 min all-out) offer practical value, the 2000 m time or average power remains the primary reference outcome for performance synthesis.

VO_2_max and PPO emerged as the strongest physiological variables associated with rowing performance. Both were primarily assessed using the INCR test, whereas the 2000 m test, the most frequently employed protocol (*n* = 24 of 30 studies), measured AP or total time. Variables obtained from INCR demonstrated very large correlations with 2000 m performance, highlighting their relevance as predictors of rowing success. In elite rowers (Class A), male VO_2_max values exceeded 5.30 L·min^−1^, showing near-perfect correlations with 2000 m AP (r = 0.93–0.99) [1,9,34,36]. Sub-elite athletes (Class B) presented intermediate values with greater variability; for instance, male VO_2_max ranged from 4.19 to 5.68 L·min^−1^ [7,8,13,14,15,18,19], maintaining strong correlations with 2000 m performance (r = 0.76–0.84). Female athletes in this category exhibited slightly lower VO_2_max (3.5–3.8 L·min^−1^) but retained very large correlations (r = 0.83) [22], consistent with known sex-based physiological differences [50]. Among recreational rowers (Class C), correlations remained large to very large: men r = 0.61–0.88 [10,11,18,20,26,38,40,42,43], women r = 0.87–0.92 [12,16], and mixed groups r = 0.88–0.96 [23,37,41]. These findings emphasize the central role of VO_2_max and PPO obtained from the INCR test as universal predictors of rowing capacity, with a clear and consistent link to performance in the 2000 m test. The largest correlations between VO_2_max and 2000 m performance in elite rowers (r = 0.93–0.99) compared to recreational athletes (r = 0.61–0.88) likely result from both physiological and methodological factors. In elite cohorts, smaller inter-individual variability in VO_2_max and stricter testing protocols enhance correlation strength through reduced inter-individual variability (i.e., range restriction) [3,19]. Moreover, elite performance depends more directly on maximal aerobic capacity and stroke efficiency [1,51], whereas in recreational athletes, technique and body composition contribute more to performance variance. Consequently, VO_2_max–performance correlations should be interpreted with caution in non-elite populations. This pattern is consistent with meta-analytic evidence showing that homogeneity of samples enhances effect sizes in correlation studies [52].

In terms of PPO obtained in the INCR test and AP during the 2000 m test showed a descending trend across elite, sub-elite, and recreational levels. Peak power obtained in the INCR test is a variable that also has a large correlation with AP over 2000 m, so it can be considered a predictor of performance. Among Class B male athletes, peak power reached up to 441 W, while AP during the 2000 m test peaked at 463 W (r = 0.92) [7,8,13,14,15,18,19]. These values are consistent with what would be expected in rowers who possess a solid training base but have not yet reached the physiological benchmarks of top international competitors. As anticipated, female athletes in this group exhibited lower values, with peak power reaching 285 W and mean 2000 m test power at 284 W (r = 0.88) [22]. However, these outcomes remain within the range reported in studies involving similarly trained female rowing populations. AP during the 2000 m test was substantially lower compared to the higher-level groups, with male values ranging between 278 and 382 W [10,11,18,20,26,38,40,42,43,47], and a single female value recorded at 245 W [12].

These findings align with prior research showing that rowing performance scales predictably with competitive level and training background [17,19]. Performance indicators such as VO_2_max and 2000 m AP have been shown to correlate strongly with years of structured training and exposure to high-intensity workloads [20]. Taken together, these findings reinforce the central role of VO_2_max and PPO as key determinants of rowing performance. The progressive variation in these variables across competitive levels underscores their sensitivity to training status and athletic development. Moreover, the observed sex-based differences align with existing literature, showing lower absolute capacities in female athletes, while relative efficiency may remain comparable [50]. To develop a more comprehensive understanding of performance in rowing, it is essential to consider not only maximal aerobic capacity but also additional physiological and biomechanical variables. Previous studies have highlighted the importance of factors such as anaerobic threshold, technical proficiency, and body composition in shaping rowing outcomes [49,50]. To summarize, the main physiological variables associated with 2000 m rowing performance, along with their definitions, correlations, advantages, and limitations, are presented in Table 9.

### 4.3. Main Findings

The main findings are summarized below, consistent with the primary research objectives. Short-duration tests—particularly the 20 s and 60 s tests—demonstrated the greatest relevance for predicting 2000 m performance [13,14,15]. Among the physiological variables, VO_2_max [1,19,22,23], power at the anaerobic threshold [13,14,15,19], and peak power during the INCR test showed the strongest associations [34]. Additional variables relevant to rowing performance include blood lactate concentration and estimates of CP or CV [7,26,39]. Directly measured VO_2_max via gas analysis remains the gold-standard marker of aerobic capacity, showing high to nearly perfect correlations with 2000 m performance (r = 0.83–0.99) in elite and sub-elite rowers, though correlations are weaker in recreational populations (r = 0.61–0.88). While traditionally measured via gas analysis, Cheng et al. [26] reported that the 3 min all-out test shows good test–retest reliability for estimating CP (r = 0.75), whereas its concurrent validity against directly measured VO_2_max via breath-by-breath gas analysis is only moderate (r = 0.66). Accordingly, the 3 min all-out test should be viewed as a practical but potentially imprecise alternative. PPO measured during INCR test, whether continuous or discontinuous, also correlates highly with rowing performance (r = 0.84–0.99) [13,14,15,19]. This variable reflects an athlete’s capacity to produce maximal power under increasing fatigue. It can be easily assessed with rowing ergometers and does not require specialized equipment, though it is influenced by the athlete’s motivation and test standardization. Blood lactate concentration, particularly the power output at the anaerobic threshold (4 mmol·L^−1^), is widely used for determining training zones and predicting performance (r = 0.83–0.92) [12,20]. However, correlations drop significantly when peak lactate values are considered (r = 0.27) [1], limiting its predictive utility beyond the threshold. Despite its reliability, lactate testing is invasive and requires skilled personnel for sample collection and analysis, which can be a logistical limitation.

CP and CV are promising indicators of maximal sustainable rowing power. They can be calculated using three to four maximal efforts of different duration, but the 3 min all-out test provides a simpler and equally reliable estimate. Although typically requiring multiple sessions, adaptations such as the 3 min all-out test [26] allow for practical field-based estimation. CP has shown strong correlations with MLSS and with 2000 m AP (r = 0.88) [39]. However, recent research by Dwyer et al. [25] found that CP showed a stronger correlation with rowing performance (male: r = −0.81 and female r = −0.68) than the lactate-based methods, confirming its criterion validity as a robust indicator of sustainable performance in rowing. While the test–retest reliability of CP protocols has been less extensively reported than their predictive validity, CP nonetheless provides a valuable and time-efficient tool for monitoring endurance capacity. Its strong association with key physiological thresholds supports its practical relevance, particularly when laboratory gas-analysis assessments are not feasible. Overall, both VO_2_max and peak power output consistently show large to nearly perfect correlations with 2000 m ergometer performance (r = 0.83–0.99), confirming their predictive value across competitive levels. While some demand advanced equipment and expertise, others—such as estimated VO_2_max or CP—present efficient, accessible, and field-applicable alternatives with growing scientific support.

Several studies have investigated alternative rowing tests to the standard 2000 m race to assess performance. Common distance tests include 100 m, 500 m, 1000 m, and 6000 m. For instance, da Silva et al. [8] found a strong correlation (r = 0.74) between AP in the 100 m test and 2000 m performance. The 500 m (r = 0.65) and 1000 m (r = −0.86) [7] tests were used to estimate CP and its association with MLSS. Holmes et al. [16] highlighted the relevance of using “split” (average 500 m pace) as a performance metric. In their study, the split correlated more strongly with CP (r = −0.99) than AP (r = −0.62) during the 2000 m test. Lastly, the 3 min all-out test proved to be a time-efficient method to determine CP. The AP from the final 30 s showed a large correlation with CP [26] and nearly perfect correlation with 2000 m AP [16], making it a valuable tool for in-season monitoring. The 6000 m test on the rowing ergometer, although less commonly used, shows strong potential as a field-based indicator of aerobic capacity. Mikulic [9] reported a strong correlation between power output at VO_2_max during an INCR discontinuous test and the power achieved in the 6000 m ergometer test (r = –0.73; *p* < 0.01), along with a moderate association with total completion time (r = –0.48; *p* < 0.05). These findings support its value for performance profiling when laboratory assessments are not feasible. Very short maximal tests, such as the 20 s and 60 s tests, also yielded nearly perfect correlations with 2000 m AP over: (60 s: r = −0.94 [15]; 20 s: r = −0.92 to −0.95) [13,14]. The Wingate test, adapted from cycling, showed large correlations with 2000 m (r = 0.79–0.87) [10,11,12] and 1500 m (r = 0.83) [11] performance in Class C and junior rowers, respectively [10,11,12]. This suggests it may be particularly reliable for youth athletes, who rely more on power in shorter races. In anticipation of the Los Angeles 2028 Olympic Games, it would be interesting to consider some of these variables or tests, since the competition distance will be reduced to 1500 m [53].

One of the most important aspects was the comparison between HWT and LWT rowers. When both groups were analyzed, LWT rowers recorded lower VO_2_max (12%), lower peak power (11%), and AP in a 2000 m test (12%) than HWT [19]. HWT rowers can produce more power due to greater weight and muscle mass than LWT rowers. Ingham et al. [1] were the only researchers who analyzed both HWT and LWT male and female athletes within a single study. Although detailed data were not reported, they concluded that the HWT group exhibited higher peak power in the 5- and 7-maximal tests, with this variable showing a nearly perfect correlation (r > 0.90) with 2000 m performance. Similarly, Nevill et al. [17] found that peak power achieved over five maximal strokes was strongly correlated with 2000 m test speed, particularly when both sexes were considered together (men: r = 0.82; women: r = 0.69; all: r = 0.94). When comparing the performance of men and women, women produced 33% less peak power in an INCR test, 34% less AP in a 20 min test [44] and 21% less VO_2_max [37]. This performance gap may be partially explained since men have a greater relative muscle mass, which allows for greater force production and greater aerobic capacity due to higher hemoglobin concentrations [53].

An additional consideration is the correlation coefficient and protocol design. Despite the correlation coefficient found in the studies, the strength of these relationships appears to depend on the competitive level. Among recreational rowers, sample heterogeneity and incomplete reporting of training history or rowing experience limit the interpretability of findings. In contrast, studies involving elite rowers generally reveal nearly perfect correlations (r > 0.90) between physiological variables and 2000 m performance. Among the 34 included studies, only 20 (59%) explicitly reported drag factor settings, highlighting a critical gap in methodological transparency that may contribute to heterogeneity in reported outcomes. Bourdon et al. [34] individualized the DF for different categories (male and female, LWT and HWT), while other studies, such as Cheng et al. [26], standardized DF at its maximum setting, whereas some allowed rowers to self-select the DF [21,47]. The rowing ergometer also requires calibration for airflow resistance, since lower damper settings increase drag resistance. Standardized procedures are therefore essential to ensure comparability of results across contexts. Variability in drag factor (standardized vs. individualized vs. self-selected) likely contributes to heterogeneity; future studies should predefine and report drag factor to improve comparability.

### 4.4. Practical Applications and Future Directions

The findings of this review highlight several promising directions for future research. First, the refinement and validation of short-duration tests could significantly enhance the efficiency of performance assessment in rowing. These protocols provide reliable data in a short time frame and induce minimal fatigue, making them particularly suitable for frequent monitoring in elite athletes [16,26]. The 3 min all-out test has shown strong correlations with both CP and VO_2_max, offering a practical alternative to the traditional INCR test, which usually demands longer durations of up to 20 min [54]. Further studies should explore whether CP estimates derived from this protocol are more accurate in elite rowers compared to those in recreational athletes, given the stronger associations observed in high-performance samples [1,19]. Moreover, considering the upcoming change in Olympic rowing distance from 2000 m to 1500 m, it is essential to investigate how this shift will influence the physiological demands of the sport. Preliminary evidence suggests an increased contribution of anaerobic metabolism and peak power output under the new format [45], reinforcing the relevance of CP and short-duration tests for performance profiling and training adaptation. From a practical perspective, short-duration ergometer protocols such as the 20 s, 60 s, and 3 min all-out tests provide valuable tools for monitoring training adaptations in rowers. These tests can be implemented regularly during the competitive season because they impose a lower physiological and psychological load than maximal 2000 m trials. Their strong correlations with 2000 m AP and CP suggest that they can serve as valid surrogates for assessing both aerobic and anaerobic performance capacities. Furthermore, the use of standardized ergometer settings facilitates reliable comparisons over time and across athletes, supporting evidence-based decision-making in load adjustment, periodization, and talent identification.

## 5. Conclusions

This systematic review confirms that ergometer tests are essential tools for evaluating rowing performance and guiding training monitoring. Among the different protocols, the 2000 m test remains the most widely studied and applied, serving as the main reference for estimating competitive capacity. The physiological variables with the strongest predictive power are VO_2_max, PPO, and power at lactate threshold, underscoring their central role in characterizing rowers’ profiles. However, the high physiological stress associated with the 2000 m test limits its practicality for frequent assessments, as it induces considerable fatigue. In this context, alternative protocols such as the 3 min all-out test emerge as valid and time-efficient options, enabling estimation of VO_2_max and CP while allowing repeated monitoring during the season. Shorter maximal efforts (20 s and 60 s tests) also provide useful alternatives to track rowing capacity with minimal disruption to training. Looking ahead, the transition to 1500 m reinforces the need to validate adapted testing protocols. Finally, despite notable advances, the literature remains limited by a lack of standardized methodologies and insufficient focus on female rowers and elite competitors. Because 72% of the analyzed sample comprised male participants and only 6 of 34 studies focused exclusively on female rowers, these findings should be interpreted primarily in the context of male performance, with cautious extrapolation to female populations. Future research should specifically address female rowers and examine whether physiological predictors of 2000 m performance differ by sex. Optimizing the integration of reliable, time-efficient ergometer protocols into routine practice is therefore crucial for individualizing athlete preparation and meeting evolving competitive demands.

## Figures and Tables

**Table 1:** Risk of Bias Assessment criteria [30].

Criteria	Definition	Scoring		
		0	1	2
Peer Reviewed	Study published in a peer-reviewed journal	No	Yes	
Number of participants	Number of participants included in study findings	<5	5–50	>50
Population defined	Age, Sex, Sport, Participation level and Experience stated	No	Partly	Yes
Methodology clearly defined	Methodology is clear and detailed enough manner for it to be repeated	No	Partly	Yes
Statistical test reported	A statistical test or significance for each correlation coefficient is discussed	No	Yes	

**Table 2:** Distribution of rowers by competitive class and sex.

Competitive Class	Male (*n*)	Female (*n*)	% of Total Male	% of Total Female	Reference
Class A	116	67	18%	27%	[1,9,25,34,35]
Class B	225	70	34%	28%	[7,8,13,15,19,22,36]
Class C	316	115	48%	45%	[10,11,12,13,16,20,23,26,37,38,39,40,41,42,43,44,45,46]

**Table 3:** Participant characteristics and aim(s) of the studies on the physical evaluation with rowing ergometer.

Author	Year	Number of Participants		Rowers Classification	Age	Aim(s) of the Study
		Male	Female			
Cosgrove et al. [20]	1999	13		Class C	19.9 ± 0.6	Examine the relationship between selected physiological variables and rowing performance as determined by a 2000 m time-trial.
Gillies and Bell [37]	2000	10	22	Class C	22 ± 5	Examine the physiological requirements of a simulated 2000 m rowing trial to determine if this relationship differs between genders.
Ingham et al. [1]	2002	19 HWT and 4 LWT	13 HWT and 5 LWT	Class A	25.8 ± 4.1	Examine the aerobic and anaerobic determinants of performance during 2000 m of rowing on an ergometer.
Riechman et al. [12]	2002		12	Class C	21.3 ± 3.6	Develop a model to predict 2000 m indoor rowing performance time from sprint performance.
Bourdin et al. [19]	2004	31 HWT		Class B	23 ± 3.7	Test the hypothesis that power peak is an overall index of rowing performance and study the influence of selected physiological variables.
		23 LWT			22.6 ± 3.7	
Messonnier et al. [33]	2004	9		Class A	22 ± 3	Relate rowing performance and associated physiological variables and investigate the specificity of the training intensity on these variables.
		12		Class C		
Bourdon et al. [34]	2009	2	8	Class A	20.9 ± 2.1	Determine whether INCR exercise and a 2000 m time trial could be combined into a single test without affecting the validity of the blood lactate threshold and/or performance data collected.
Mikulic [9]	2009	25		Class A	22.2 ± 4.8	Examine the anthropometric and metabolic determinants of performance during 6000 m of rowing on an ergometer.
Shimoda et al. [38]	2009	16		Class C	20.7 ± 0.9	This prompted us to suppose dependence on stroke consistency, aerobic capacity, leg extension power, and rowing performance.
Izquierdo-Gabarren et al. [18]	2010	24		Class B	28 ± 5	Examine which one of the performance factors would be able to differentiate rowers at different standards in traditional rowing and determine the best predictors of traditional rowing performance.
		22		Class C	23 ± 4	
Kendall et al. [39]	2011		19	Class C	19.7 ± 1.4	Assess the critical velocity (CV) test as a means of predicting 2000 m performance and to study the effect of selected physiological variables.
Cheng et al. [26]	2012	18		Class C	17.7 ± 1.9	Determine the test–retest reliability of the 3 min all-out rowing test and the differences between traditional CP tests.
Ingham et al. [23]	2013	4 HWT and 6 LWT	4 HWT and 4 LWT	Class C	23.3 ± 3.1	Examine the relationship between parameters derived from both an INCR test in relation to 2000 m ergometer rowing performance.
Akça [10]	2014	38		Class C	20.1 ± 1.2	Develop different regression models to predict 2000 m rowing ergometer performance.
Cataldo et al. [13]	2015	20		Class C	15.2 ± 1.3	Evaluate the relationship between the AP during the 20 s all-out test rowing ergometer test and the 2000 m indoor rowing performance.
Otter et al. [40]	2015	24		Class C	23 ± 1	Assess the predictive value of the Submaximal Rowing Test on 2000 m ergometer rowing time in competitive rowers
Maciejewski et al. [11]	2016	14		Class C	15.3 ± 0.6	Determine whether anaerobic performance assessed from a 30 s all-out test could account for the 1500 m rowing performance.
Bourdin et al. [22]	2017		43 HWT	Class B	21.9 ± 3.7	Point out the predictive factors of physical performance in high-level female rowers and evaluate whether the relative influence of these factors is like that observed in males.
			27 LWT		20.6 ± 2.9	
Cerasola et al. [15]	2020	15		Class B	15.7 ± 2.0	Develop different regression models to predict 2000 m rowing indoor performance time using VO_2_max and AP established during a 60 s all-out test.
Holmes et al. [16]	2020		31	Class C	20.2 ± 1.1	Examine the associations of CP from a 3 min all-out row test and peak power from the 1-Stroke with VO_2_peak, Wingate Test, 6000 m and 2000 m rowing ergometer test.
Turnes et al. [41]	2020	16	3	Class C	25.5 ± 10.6	Identify the relationship between the AP of 2000 m rowing ergometer performance with the PPO obtained during an INCR test and verifying the possibility of achieving VO_2_max during a 2000 m time trial and using the AP of this test.
Da Silva et al. [8]	2021	12		Class B	15.9 ± 1.0	Develop a mathematical model capable of predicting 2000 m performance from a 100 m maximal effort test.
Jensen et al. [47]	2021	7		Class A	25.4 ± 5.2	(1) Compare VO_2_max measured in a 2000 m test and a continuous INCR test, (2) determine the linear relationship between AP during 2000 m and VO_2_max, (3) and determine the linear relationship between maximal PPO measured in a continuous INCR test and VO_2_max.
Possamai et al. [7]	2021	27		Class C	26 ± 13	(1) Compare the intensities of maximal lactate steady state (MLSS) and CP in trained rowers, (2) describe the relationship of MLSS with performances of 500 m, 1000 m, 2000 m, and 6000 m rowing ergometer time-trial tests
		14		Class B		
Cerasola et al. [14]	2022	17		Class B	15.8 ± 2	Investigate the relationship between the fixed-time 20 s and 60 s all-out tests and the fixed-distance 2000 m indoor rowing performance.
Huerta Ojeda et al. [42]	2022	12		Class C	20.3 ± 1.6	Describe and analyze the kinetics of ventilatory and mechanical parameters on the rowing ergometer.
Huerta Ojeda et al. [43]	2022	12		Class C	20.3 ± 1.6	The main objective of this study was to determine the validity and reliability of the 6 min all-out test as a predictor of Maximal Aerobic Power (MAP).
Mazza et al. [21]	2023		10	Class A	23.3 ± 2.8	Develop an equation that can be used to estimate VO_2_max in female rowers using the same INCR test method.
			20			
McGrath et al. [44]	2023	11	20	Class C	25.5 ± 3.2	The m-FTP equation could accurately predict rowing Functional Threshold Power (r-FTP) data in a more heterogeneous cohort of club-level male and female rowers
Astridge et al. [45]	2023	18		Class C	16.7 ± 0.4	Compare the energetic contribution to, and pacing strategies adopted over, maximal 2000 m and 1500 m ergometer rowing performance
Clark et al. [35]	2025	30	13	Class A	22.9 ± 3.8	Determine the reliability and usefulness of the 6000 m rowing ergometer training-based test to evaluate its suitability as a monitoring tool in high-level rowers
Dwyer et al. [25]	2025	20	18	Class A	NR	(1) determine the level of agreement between CP and Lactate threshold in elite-level rowers, (2) describe and compare the relationship between 2000 m rowing time-trial performance and lactate threshold and 2000 m rowing performance and CP, (3) determine the accuracy with which the CP model can predict 2000 m rowing performance
House et al. [46]	2025	23		Class C	14.89 ± 0.60	Better understand the associations that specific measures of maximal and rapid force production may have with rowing ergometer performance in male adolescent rowers

Abbreviations: HWT, heavyweight; LWT, lightweight; NR, not reported.

**Table 4:** Key physiological variables by competitive level and sex.

Competitive Level	Sex	VO_2_max (L·min^−1^)	PPO (INCR) (W)	AP 2000 m (W)	Reference
Class A (Elite)	Men	5.33–5.53			[1,9,34,36]
	Women				
Class B (Sub-elite)	Men	4.19–5.68	308–441	235–463	[7,8,13,14,15,18,19]
	Women	3.50–3.80	266–285	259–284	[22]
Class C (Recreational)	Men	4.07–5.30	234–350	275–382	[10,18,20,26,38,40,42,47]
	Women	3.18		245	[12,16,23,37,41]

**Table 5:** Distance and time test in rowing ergometer and the corresponding time or power variables.

Author	Test				Power in W (Mean ± SD)				Time in s (Mean ± SD)	
Akça [10]	(1) 2000 m	(2) Wingate Test			(2) 638 ± 41.80				(1) 398.50 ± 20.11	
Astridge et al. [45]	(1) 2000 m	(2) 1500 m			(1) 324 ± 24	(2) 341 ± 29				
Bourdin et al. [19]	(1) 2000 m				(1) 462.9 ± 36.8					
Bourdin et al. [22]	(1) 2000 m				(1) 275 ± 32					
Bourdon et al. [34]	(1) 2000 m								(1) 430.0 ± 7.3	
Cataldo et al. [13]	(1) 2000 m	(2) 20 s			(2) 501.7 ± 113.0				(1) 425.0 ± 25.8	
Cerasola et al. [14]	(1) 2000 m	(2) 60 s			(2) 476.1 ± 91.0				(1) 417.1± 21.8	
Cerasola et al. [15]	(1) 2000 m	(2) 20 s	(3) 60 s		(2) 525.1 ± 113.7	(3) 476.1 ± 91.0			(1) 418.5 ± 23.1	
Cheng et al. [26]	(1) 3 min all-out				(1) EP: 269 ± 39					
Clark et al. [47]	(1) 6000 m	(2) 2000 m				(2) 370 ± 71			(1) 1341 ± 86	
Cosgrove et al. [20]	(1) 2000 m				(1) NR					
Da Silva et al. [8]	(1) 2000 m	(2) 100 m			(1) 235.9 ± 29.0	(2) 376.9 ± 62.7				
Dwyer et al. [25]	(1) 2000 m	(2) 6000 m	(3) 500 m		(1) 313 ± 24				(2) 1160 ± 20	(3) 78 ± 2
Gillies and Bell [37]	(1) 2000 m								(1) 475.1 ± 7.4	
Holmes et al. [16]	(1) 2000 m	(2) 6000 m	(3) 3 min all-out		(3) EP: 232.61 ± 31.27				(1) 444.96 ± 10.83	(2) 1432.73 ± 35.71
House et al. [46]	(1) 2000 m				(1) 178.17 ± 52.48					
Huerta Ojeda et al. [42]	(1) 2000 m								(1) 431.4 ± 12.7	
Huerta Ojeda et al. [43]	(1) 6 min				(1) 289.83 ± 20.91					
Ingham et al. [1]	(1) 2000 m				(1) NR					
Ingham et al. [23]	(1) 2000 m				(1) 346.5 ± 75.5					
Izquierdo-Gabarren et al. [18]	(1) 2000 m	(2) 6 min			(2) 272.69 ± 30				(1) 386 ± 10.47	
Jensen et al. [47]	(1) 2000 m				(1) 333 ± 69					
Kendall et al. [39]	(1) 2000 m								(1) 467.6 ± 17.8	
Maciejewski et al. [11]	(1) 1500 m	(2) Wingate Test			(1) 279.7 ± 49.1	(2) 429.2 ± 92.				
Mazza et al. [21]	(1) 2000 m				(1) NR					
McGrath et al. [44]	(1) 6 min				(1) 230 ± 64					
Messonnier et al. [33]	(1) 2000 m				(1) MP: 432 ± 12					
Mikulic [9]	(1) 6000 m								(1) 1195.4 ± 36.1	
Otter et al. [40]	(1) 2000 m								(1) 389 ± 14	
Possamai et al. [7]	(1) 2000 m	(2) 6000 m	(3) 500 m	(4) 1000 m	(1) 317 ± 38	(2) 258 ± 28	(3) 499 ± 47	(4) 372 ± 40		
Riechman et al. [12]	(1) 2000 m	(2) Wingate Test			(2) 368 ± 60.0				(1) 466.8 ± 12.3	
Shimoda et al. [38]	(1) 2000 m								(1) 409.3 ± 1.2	
Turnes et al. [41]	(1) 2000 m				(1) 284.2 ± 49.9					

Abbreviations: EP, End Power (last 30 s AP); NR, not reported.

**Table 8:** Number and distribution of tests included in the present review.

Type of Test		
Distance [2000 m]		*n* = 30 [23]
INCR test	Discontinuous	*n* = 16
	Continuous	*n* = 13
Time		*n* = 12
Maximal Strokes		*n* = 4

**Table 9:** Summary of key physiological variables associated with 2000 m rowing performance: definitions, advantages, and limitations.

Physiological Variable	Definition/Description	2000 m Performance	Advantages	Limitations
VO_2_max	Maximal oxygen uptake (L·min^−1^) during INCR test using gas analysis.	Very large to nearly perfect (r = 0.76–0.99).	Gold standard for aerobic fitness; high predictive power; validated across populations.	Requires expensive equipment and lab setting; time-consuming; effort-dependent.
	Estimated from 3 min all-out test (non-invasive).	Strong agreement with gas-based VO_2_max.	Time-efficient; low-cost; suitable for field testing.	Less validated; may not replace direct gas analysis in elite settings.
Peak Power Output (INCR)	Highest power (W) during INCR test to exhaustion (continuous or discontinuous).	Large to nearly perfect (r = 0.84–0.99).	Easy to measure; non-invasive; correlates with VO_2_max and 2000 m performance.	Requires maximal effort and strict protocol standardization.
Blood Lactate Concentration	Measured during INCR test at anaerobic threshold (4 mmol·L^−1^); reflects aerobic/anaerobic transition.	Large to very large at threshold (r = 0.83–0.92); poor peak lactate (r = 0.27).	Enables individualized training zones; widely used in endurance sports.	Invasive (blood sampling); limited utility beyond threshold; requires biochemical analysis.

## Data Availability

The dataset is available on reasonable request from the corresponding author.

## Abbreviations

The following abbreviations are used in this manuscript:

AP	Average Power
CP	Critical Power
CV	Critical Velocity
FISA	Fédération Internationale des Sociétés d’Aviron
INCR	Incremental
PPO	Peak Power Output
VO_2_max	Maximal oxygen uptake
W	Watts
